# How well do HapMap SNPs capture the untyped SNPs?

**DOI:** 10.1186/1471-2164-7-238

**Published:** 2006-09-19

**Authors:** Erwin Tantoso, Yuchen Yang, Kuo-Bin Li

**Affiliations:** 1 Bioinformatics Institute, 30 Biopolis Street, #07-01 Matrix, 138671, Singapore; 2 Bioinformatics Center, National Yang-Ming University, Taipei, 112, Taiwan

## Abstract

**Background:**

The recent advancement in human genome sequencing and genotyping has revealed millions of single nucleotide polymorphisms (SNP) which determine the variation among human beings. One of the particular important projects is The International HapMap Project which provides the catalogue of human genetic variation for disease association studies. In this paper, we analyzed the genotype data in HapMap project by using National Institute of Environmental Health Sciences Environmental Genome Project (NIEHS EGP) SNPs. We first determine whether the HapMap data are transferable to the NIEHS data. Then, we study how well the HapMap SNPs capture the untyped SNPs in the region. Finally, we provide general guidelines for determining whether the SNPs chosen from HapMap may be able to capture most of the untyped SNPs.

**Results:**

Our analysis shows that HapMap data are not robust enough to capture the untyped variants for most of the human genes. The performance of SNPs for European and Asian samples are marginal in capturing the untyped variants, i.e. approximately 55%. Expectedly, the SNPs from HapMap YRI panel can only capture approximately 30% of the variants. Although the overall performance is low, however, the SNPs for some genes perform very well and are able to capture most of the variants along the gene. This is observed in the European and Asian panel, but not in African panel. Through observation, we concluded that in order to have a well covered SNPs reference panel, the SNPs density and the association among reference SNPs are important to estimate the robustness of the chosen SNPs.

**Conclusion:**

We have analyzed the coverage of HapMap SNPs using NIEHS EGP data. The results show that HapMap SNPs are transferable to the NIEHS SNPs. However, HapMap SNPs cannot capture some of the untyped SNPs and therefore resequencing may be needed to uncover more SNPs in the missing region.

## Background

The abundance of single nucleotide polymorphism (SNP) in the human genome sequence offers a way for genetic association studies. Association studies usually involve comparing the allele frequency of a particular SNP in unrelated controls and cases (patients)[1]. A SNP that is observed at a higher incidence in cases compared to controls can be shown to be significantly associated with the phenotype, which is dependant on the panel sizes and the measure of the difference in observed allele frequencies between the panels. However, a statistically significant association of a SNP with a phenotype does not necessarily indict the SNP as a causal variant, rather it could be that the observed SNP is in linkage disequilibrium (LD) with the causal variant [1-4]. Therefore, involving the causal SNP or the marker SNP that is in LD with the causal variant will be important to detect disease association. Ideally, we can include all the SNPs identified in the human genome sequence to perform disease association studies. However, due to the abundance of SNPs in the human genome, it becomes impractical to genotype each one of them for association studies. Therefore, the knowledge of haplotype structure and linkage disequilibrium [5-8] provide a cost effective way to reduce the number of SNPs by exploiting the correlation between them. Several algorithms have been proposed in order to choose tag SNPs, i.e. the subset of SNPs which can capture all the other SNPs [9-16].

As the whole-genome association studies become important to understand the underlying variation that leads to human diseases, the International HapMap Project [17-19] was launched to provide a catalog of human genetic variation in four different populations, i.e. 30 trios of CEPH (the US Utah population with Northern and Western European ancestry), 45 unrelated samples of CHB (Han Chinese in Beijing, China), 44 unrelated samples of JPT (Japanese in Tokyo, Japan) and 30 trios of YRI (Yoruba people in Ibadan, Nigeria). The aim of the International HapMap project is to identify the common patterns in DNA sequence variation and the correlation between them. Therefore, the catalog can be used as a reference to choose tagSNPs for association studies.

With the almost complete human genetic variation map by HapMap project, a number of studies have looked into the utility of this catalog as a reference panel for association study. Are the tagSNPs chosen from the HapMap reference panel transferable to the population in other geographical region? Are the HapMap SNPs able to act as a reference panel and show concordance to other close populations? de Bakker et. al [20] addressed the issue of tag SNP transferability by genotyping 2783 SNPs across 61 genes involved in DNA repair. Their results showed that common variation can be captured robustly in the non-African samples with little loss of power. Montpetit et. al[21] evaluated the performance of the tagSNPs derived from HapMap in the Caucasian population. Their result showed that the tagSNPs selected from HapMap CEU panel capture most of the common variation in the Estonian sample well. In order to assess whether HapMap SNPs are applicable to other closely related population, Ribas et. al. [22] evaluated the HapMap SNP data transferability with CEU as a reference panel in the Spanish population. Their results showed that HapMap SNPs data are applicable for the Spanish population. Similarly, Willer et. al. [23] demonstrated that the HapMap CEU panel can be used as the reference panel for tag SNP selection in the Finnish individuals. The above studies have shown that tagSNPs are transferable to other population and HapMap SNPs demonstrate concordance with the closely related population. Nevertheless, are HapMap SNPs or tagSNPs able to capture the untyped SNPs which may not be genotyped in the HapMap reference panel?

In this work, we attempt to address the question of whether HapMap SNPs are sufficient to capture most of the variation and untyped SNPs in the human genes by using the SNPs identified by National Institute of Environmental Health Sciences (NIEHS SNPs)[24,25]. We choose NIEHS EGP SNPs to assess the performance of HapMap SNPs because NIEHS SNPs are the result of gene resequencing and therefore are more comprehensive than the HapMap SNPs. Using NIEHS SNPs enable us to perform computational analysis without performing any genotyping. This analysis will be valuable as to understand the comprehensiveness of HapMap SNPs. By using HapMap as a reference panel, first we seek to determine whether HapMap SNPs are transferable to the NIEHS dataset. Then we test whether the SNPs chosen from HapMap will be able to capture the untyped SNPs in the NIEHS. We observed that HapMap SNPs performed very well in some genes, but are unable to capture the untyped variants in most of the genes. Having observed the performance of HapMap SNPs, we identify that the SNP density and association among the SNPs in HapMap play an important role in determining the performance of SNPs in the gene. Therefore, we provide general guidelines on how to determine if the HapMap SNPs in a gene are comprehensive enough as a reference for association studies.

## Results

### Not all SNPs genotyped in HapMap are identified in NIEHS

Figure 2 (Methods Section) illustrates the two conditions of the data, i.e. set A and set B. Table 1 shows the number of genes that is categorized as the first (set A) or the second (set B) condition and also reports the total number of genes analyzed in each population.

### HapMap data are transferable to the NIEHS SNPs

We used the genes in set B for transferability assessment. TagSNPs are chosen from HapMap data using pairwise-r^2 ^method with the parameter r^2 ^≥ 0.80. The tagSNPs are then applied to the NIEHS data and the performance is measured. Table 2 shows that the HapMap data are transferable to the NIEHS SNPs with coverage of more than 95%.

### HapMap data are not robust enough to capture the untyped SNPs

To assess the robustness of HapMap SNPs, the genes in set B are used for analysis. Table 3 shows that the HapMap SNPs are not robust enough to capture the untyped SNPs with a threshold of r^2 ^≥ 0.80. It can be observed that the European and Asian population have similar performance with coverage of approximately 50% only. Expectedly, the HapMap SNPs for African population show the worst performance with coverage of approximately 30% only.

### NIEHS SNPs is a better reference panel for gene-based association study

As shown in Figure 2A, not all SNPs in HapMap are identified in NIEHS. We use the genes in set A to determine which dataset is a better reference panel for gene-based association study. The overlapped SNPs are used and the ability of these overlap SNPs to capture other SNPs in the dataset are assessed. Table 4 shows that the number of SNPs identified in NIEHS is much greater than the SNPs genotyped in HapMap Project. Using the HapMap-NIEHS overlapped SNPs; we indeed observe that the coverage is low in the NIEHS population as compared to the HapMap population.

### SNPs density and association among SNPs determine the ability of HapMap SNPs to capture untyped SNPs in NIEHS

**Figure 1:**
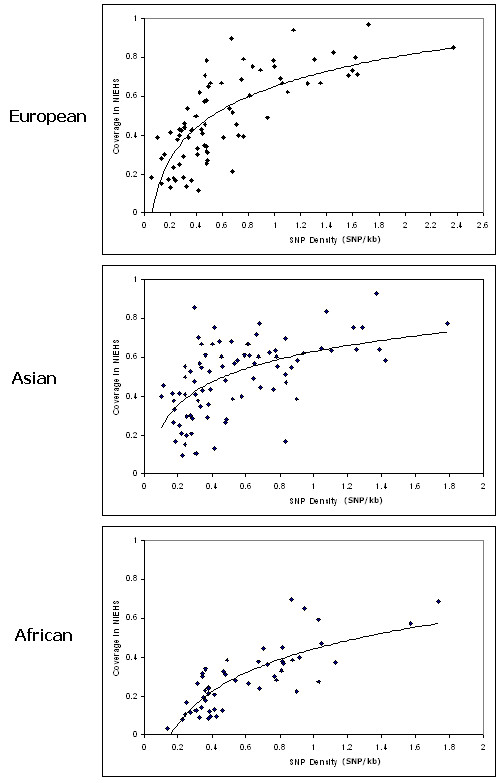
**Coverage versus HapMap SNP density in three populations**. The coverage increases with higher SNP density. High coverage is observed in some of the genes with low SNP density in Asian and European population. The reason may be due to the high association among SNPs in these regions.

We observe that SNP density and the association among SNPs determine the ability of using HapMap SNPs to capture the untyped SNPs. Figure 1 shows that the coverage of HapMap SNPs increases along with the SNP density. Some genes in European and Asian population have low SNP density but have high coverage. We believe that the high coverage is due to the high LD among SNPs in the European and Asian populations as compared to the African population.

## Discussion

We conducted analyses on HapMap data and determined whether the HapMap data are sufficient for association studies and able to cover most of the untyped SNPs. As a comparison, we used the NIEHS EGP SNPs to determine the performance of HapMap SNPs. We chose NIEHS EGP SNPs because the SNPs identified in NIEHS EGP are the results of resequencing. However, before further analysis can be done, due to the unequal sample size between HapMap and NIEHS dataset, we need to test whether the HapMap tagSNPs are transferable to the NIEHS populations. Table 2 shows that the HapMap tagSNPs are transferable to the NIEHS population with coverage of more than 95%. Montpetit et. al. [21] have shown that HapMap SNPs are transferable and we have confirmed their results. However, transferability of the HapMap SNPs can not ensure that the SNPs can capture other untyped SNPs. It has been proposed that SNPs that are highly associated with diseases may be due to LD between the causal SNP with the marker SNP [1-4]. Therefore if the marker SNPs used for disease association can not capture most of the untyped SNPs, we could miss important marker SNPs that are in LD with the causal SNPs.

**Figure 2:**
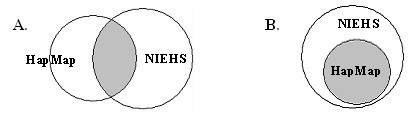
**Illustration of HapMap and NIEHS SNP**. Figure 2A represents the genes in first condition (set A), i.e. not all the SNPs in HapMap are identified in NIEHS dataset and vice versa. The shaded area is the overlapping SNPs between the two datasets. Figure 2B represents the genes in second condition (set B) where the SNPs in HapMap are all genotyped in the NIEHS dataset or the subset of SNPs in HapMap can cover all other SNPs in HapMap.

Having shown that the HapMap SNPs are transferable to the NIEHS population, we then assess whether HapMap SNPs are able to capture other untyped SNPs in the regions. Although the NIEHS SNPs are identified through resequencing, not all the regions in the genes are resequenced. In fact, some regions are skipped. Therefore, certain SNPs genotyped in the HapMap Project are not identified in the NIEHS EGP SNPs. This is the reason why we divided our analysis into two parts. The first part is for the condition where not all the SNPs inside the genes in HapMap are identified in the NIEHS (Figure 2A). We refer these genes as set A. For the genes in set A, the SNPs that are common to both dataset are chosen. These common SNPs are then used to measure the comprehensiveness of both datasets. The high coverage of the common SNPs in a particular dataset indicates that the dataset is not as comprehensive as the other one. Table 4 shows that the coverage of those common SNPs are higher in the HapMap with an approximate 80% of coverage but the performance of the common SNPs drops to approximately 50% when applied to the NIEHS dataset. The second part of our analysis applies to the condition where all the SNPs in HapMap or a subset of SNPs which can capture all other SNPs are available in the NIEHS. We refer these genes as set B. Certainly these SNPs represent 100% of the HapMap SNPs. However, when these SNPs are applied to NIEHS, we observe that the coverage is only around 50% in the European and Asian populations. The coverage is lower in the African population with only 30%.

Results show that the HapMap data are not robust enough to capture most of the untyped variants. These results are baffling since some studies have used HapMap as the reference panel to choose tagSNPs for association study. Although the overall coverage of HapMap SNPs is low, the low coverage is not applied to all genes under studied. In fact, some of the genes give a very high coverage of the untyped genes. Therefore, before HapMap data can be used as the reference panel for gene-based association study, it may be important to do a preliminary analysis of the HapMap SNPs to ensure their comprehensiveness. We attempted to identify the preliminary analysis that is necessary before concluding that HapMap SNPs are comprehensive enough for gene-based association study. It is observed that the SNP density and the association among SNPs in the region play an important role in determining the comprehensiveness of HapMap SNPs. A graph of coverage versus SNP density (the number of SNPs per kb sequence) is plotted for this purpose. Figure 1 shows that the coverage increases along with the SNP density. As expected, the African population needs higher SNPs density in order to capture most of the untyped SNPs. However, for certain genes, only a marginal SNP density will be able to produce a high coverage. This observation is clear in the European and the Asian population but not in the African population. This is probably due to the low recombination event in the European and Asian population as compared to the African population. Therefore, we suggest that the linkage disequilibrium and high association among the SNPs in the European and Asian population may be the reason behind the high coverage for low SNP density genes. Having observed the relation between SNP density and high association among SNPs towards SNPs coverage, we propose that in order to ensure that the chosen SNPs can cover the untyped SNPs; the SNP density is the major parameter to be aware of. However, if the SNP density is marginal, the LD pattern for the region may serve as an additional guidance towards the confidence that the SNPs can capture most of the untyped SNPs.

## Conclusion

HapMap SNPs have been shown to be transferable to NIEHS SNPs. However, the transferability of HapMap SNPs does not mean that they can be used to capture all other untyped SNPs. We have analyzed the ability of HapMap SNPs to capture other untyped SNPs in the NIEHS SNPs. Our results show that HapMap SNPs are not robust enough to capture the untyped SNPs. SNP density and association among SNPs in the HapMap dataset might be the explanation. Due to the limitation of using HapMap SNPs to capture the untyped variants, we suggest that resequencing may be needed to uncover more SNPs in the missing region so that researchers can be certain that tagSNPs chosen for association study are able to provide a comprehensive coverage of all the variants in the genes.

## Methods

### Dataset and population samples

The dataset used in this work come from HapMap Build 35 and NIEHS EGP SNPs as at 26 May 2006 (NIEHS SNPs. NIEHS Environmental Genome Project, University of Washington, Seattle, WA [26] [May 2006 accessed]). The dataset for the HapMap project includes 30 trios of CEPH (the US Utah population with Northern and Western European ancestry), 45 unrelated samples of CHB (Han Chinese in Beijing, China) and 30 trios of YRI (Yoruba people in Ibadan, Nigeria) population. As an assessment, the NIEHS populations include 22 Europeans (all samples are subset of HapMap CEU samples), 27 Africans (12 African Yoruban samples are subset of HapMap YRI samples) and 24 Asians (12 samples are subset of HapMap CHB samples) from the panel P2. We chose panel P2 because genes are resequenced in the population of known ethnicities, thus we can separate them based on the population and assessment can be made according to populations.

### Gene selection for analysis

As at 26 May 2006, 199 genes had been resequenced in NIEHS panel P2. The genotype data for each gene are downloaded and the corresponding chromosomal locations are identified. Using the chromosomal location identified for each gene, we downloaded the corresponding polymorphism data from The International HapMap Project website.

A gene is excluded provided either one of the following three conditions is met:

1. All the SNPs inside the gene have minor allele frequency less than 5%.

2. Multiallelic SNP appears in the gene.

3. No common SNPs between the HapMap dataset and NIEHS dataset.

The total number of genes chosen for further analysis is listed in Table 1. The list of genes chosen for analysis in each population is given in the Additional file 1.

### Categorization of SNPs into set A and set B

Figure 2 shows the two conditions when analyzing the HapMap dataset with the NIEHS EGP SNPs. The first condition (set A) is illustrated in Figure 2A where not all the SNPs in HapMap for the set of genes are identified in NIEHS. The SNPs that are common to both datasets are shown in the shaded area. The second condition (set B) is illustrated in Figure 2B where all the SNPs in HapMap for the set of genes are identified in NIEHS. In addition, some genes were initially categorized into set A, but later they were included in set B. These genes do not have all their SNPs available in the NIEHS; however, a subset of them can capture all other SNPs with r^2 ^≥ 0.80. Genes with these characteristic are considered as the set B genes. Please refer to Figure 4 for the flowchart of how genes are categorized into set A and set B.

### HapMap SNPs transferability assessment

**Figure 3:**
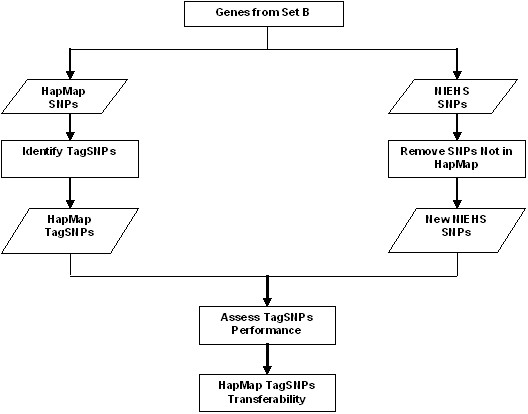
**Flowchart of HapMap SNPs transferability assessment**. Genes from set B are used for transferability assessment. For transferability assessment, SNPs in NIEHS that are not genotyped in HapMap are removed. HapMap tagSNPs are applied to the new NIEHS SNPs and the tagSNPs performance is assessed.

Figure 3 shows the work flow of assessing the HapMap SNPs transferability. Transferability is defined as the capability of SNPs in one population to be transferred to other population. In this work, transferability is limited to the same population. The idea is to ensure that the HapMap SNPs are transferable to NIEHS SNPs and the incapability of HapMap SNPs to capture untyped variants is not due to the caveat of different sample size. For HapMap SNPs transferability, only the set B (as illustrated in Figure 2B) is considered. First, we need to remove all the SNPs in NIEHS that are not genotyped in HapMap. SNPs concordance is determined based on rsID. After that, a set of tagSNPs are chosen for HapMap SNPs using Haploview ver 3.2 [15]. The set of tagSNPs are then assessed in the new NIEHS SNPs using a threshold of r^2 ^≥ 0.80. So, all other SNPs which have pairwise-r^2 ^≥ 0.80 with the tagSNPs are considered as captured. SNP transferability is performed on the identical samples, for example: HapMap CEU panel is used as the reference panel for the European sample in NIEHS SNPs, CHB panel for Asian and YRI panel for African.

### Identifying Linkage Disequilibrium for each gene

The pairwise-r^2 ^value for each pair of the SNPs is calculated for both HapMap and NIEHS SNPs using Haploview ver 3.2. Given the pairwise-r^2 ^value for each pair of the SNPs, an LD table is created for each gene. LD table is a two-dimensional array that stores the pairwise-r^2 ^values for each pair of the SNPs.

### Performance measurement

The SNPs from the reference panel are applied to the studied populations. The performance is reported as the mean-r^2^, min-r^2 ^and coverage of the reference SNPs. We use Haploview to get the pairwise-r^2 ^as stated above. For coverage measurement, the SNP is called covered if the pairwise-r^2 ^between the untyped SNP and the genotyped SNP has a pairwise-r^2 ^greater than the threshold. In this study, we use r^2 ^threshold = 0.80.

### Overall workflow

**Figure 4:**
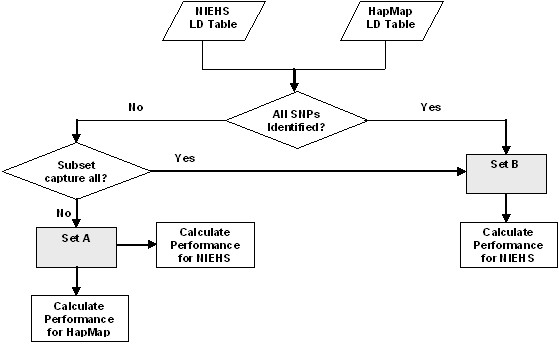
**Overall workflow of HapMap SNPs assessment**. If all the SNPs in HapMap or subset of SNPs in HapMap that can capture all other SNPs are identified in NIEHS, then classified them as set B. Otherwise, the genes are classified as set A. For the genes in set A, the performance in both NIEHS and HapMap are measured. For the genes in set B, the SNPs are assessed in the NIEHS populations.

The overall workflow is given in Figure 4. LD table is created for both HapMap and NIEHS dataset. Then the genes are categorized into the set A and set B as explained above. For the set A genes, the common SNPs for both HapMap and NIEHS datasets are used to identify the coverage in HapMap or NIEHS.

For the set B genes, the SNPs are used to identify the coverage in NIEHS.

## Authors' contributions

This work was conceptualized by KBL, ET and YY. ET and YY prepared the data and did the analysis while KBL supervised and provided guidance throughout the process. ET drafted the manuscript and all authors have read and approved the final manuscript.

## Supplementary Material

Additional File 1 **List of genes in set A and set B for each population together with the transferability assessment**. A Microsoft Excel file is provided for the list of genes in set A and set B for each population. The coverage of SNPs for each dataset is provided. Transferability assessment is included as well. Click here for file

## Figures and Tables

**Table 1:** The number of genes that is categorized as the first (set A) or the second (set B) condition

**Population**	**Set A**	**Set B**	**Total**
European	88 genes	78 genes	166 genes
Asian	81 genes	84 genes	165 genes
African	99 genes	51 genes	150 genes

The total number of genes in set A or set B is given for each population. The total number of genes analyzed in each population is given as well.

**Table 2:** Transferability assessment of HapMap SNPs

**Population**	**SNPs Transferability Performance with r**^**2 **^**≥ 0.80**			
	
	**Number of SNPs**	**Mean-r**^**2**^	**Min-r**^**2**^	**Coverage**
**European**	843	0.984	0.459	96.4%
**Asian**	693	0.982	0.410	97.1%
**African**	406	0.982	0.510	94.8%

HapMap data are used as the reference panel and the corresponding population in NIEHS is assessed. HapMap CEU panel for NIEHS European assessment; HapMap CHB panel for NIEHS Asian assessment and HapMap YRI panel for NIEHS African assessment.

**Table 3:** Performance of HapMap SNPs data for set B genes

**Population**	**Number of SNPs**	**Mean-r**^**2**^	**Min-r**^**2**^	**Coverage**
European (CEU)	3101	0.580	0.000	55.0%
Asian (CHB)	2892	0.570	0.000	50.6%
African (YRI)	2673	0.403	0.000	31.0%

The performance of HapMap SNPs data in the set B genes are given for each population. The mean-r^2^, min-r^2 ^and coverage are given as the performance measurement.

**Table 4:** Performance of HapMap-NIEHS overlap SNPs in HapMap and NIEHS dataset

	**HapMap**				**NIEHS**			
	
**Population**	**Num SNPs**	**Mean-r**^**2**^	**Min-r**^**2**^	**Coverage**	**Num SNPs**	**Mean-r**^**2**^	**Min-r**^**2**^	**Coverage**
European	3691	0.885	0.000	82.6%	6292	0.619	0.002	55.7%
Asian	3041	0.887	0.009	84.8%	5963	0.608	0.000	56.2%
African	3400	0.833	0.000	73.4%	10177	0.454	0.000	34.5%

Overlapping SNPs in both datasets are used to determine the comprehensiveness of dataset. The overlapping SNPs provide higher coverage when applied to HapMap dataset compare to NIEHS dataset. In addition, the total number of SNPs in NIEHS is much more than the total number of SNPs in HapMap.
